# NovaGenesis Applied to Information-Centric, Service-Defined, Trustable IoT/WSAN Control Plane and Spectrum Management

**DOI:** 10.3390/s18093160

**Published:** 2018-09-19

**Authors:** Antônio Marcos Alberti, Marília Martins Bontempo, José Rodrigo dos Santos, Arismar Cerqueira Sodré, Rodrigo da Rosa Righi

**Affiliations:** 1 ICT Lab, Instituto Nacional de Telecomunicações—Inatel, Av. João de Camargo 510, Centro, CEP 37540-000 Santa Rita do Sapucaí, Minas Gerais, Brazil; mariliamartins@gee.inatel.br (M.M.B.); joserodrigo@gec.inatel.br (J.R.d.S.); 2 Lab WOCA, Instituto Nacional de Telecomunicações—Inatel, Av. João de Camargo 510, Centro, CEP 37540-000 Santa Rita do Sapucaí, Minas Gerais, Brazil; arismar@inatel.br; 3 Programa Interdisciplinar de Pós-Graduação em Computação Aplicada, Universidade do Vale do Rio dos Sinos—Unisinos, Av. Unisinos 950, Bairro Cristo Rei, CEP 93022-750 São Leopoldo, Rio Grande do Sul, Brazil; rrrighi@unisinos.br

**Keywords:** cognitive radio, future Internet, information-centric network, IoT, NovaGenesis, service-oriented architecture, software-defined network, spectrum management

## Abstract

We integrate, for the first time in the literature, the following ingredients to deal with emerging dynamic spectrum management (DSM) problem in heterogeneous wireless sensors and actuators networks (WSANs), Internet of things (IoT) and Wi-Fi: (i) named-based routing to provide provenance and location-independent access to control plane; (ii) temporary storage of control data for efficient and cohesive control dissemination, as well as asynchronous communication between software-controllers and devices; (iii) contract-based control to improve trust-ability of actions; (iv) service-defined configuration of wireless devices, approximating their configurations to real services needs. The work is implemented using NovaGenesis architecture and a proof-of-concept is evaluated in a real scenario, demonstrating our approach to automate radio frequency channel optimization in Wi-Fi and IEEE 802.15.4 networks in the 2.4 GHz bands. An integrated cognitive radio system provides the dual-mode best channel indications for novel DSM services in NovaGenesis. By reconfiguring Wi-Fi/IoT devices to best channels, the proposed solution more than doubles the network throughput, when compared to the case of mutual interference. Therefore, environments equipped with the proposal provide enhanced performance to their users.

## 1. Introduction

The technology evolution has been based on diverse disruptive technologies, which now start to converge towards deeply integrating physical and virtual worlds; a trend that has been called digital transformation [1]. Disruptions are emerging in connectivity, controllability, virtualization, expressiveness, content distribution and resources management. The Internet of Things (IoT) [2] is an example of disruption that has been covering connectivity, expressiveness and resources management. Expressiveness can be defined as the ability to express meaning [3]. Connected sensors provide all kind of physical world data, generating context for services and applications. Connected actuators reflect back to physical world decisions made at software level. Things’ representatives (smart objects) act as proxies, representing physical world devices towards service level dynamic composition [4].

Other important disruptions are related to the increasing role of software in information architectures. Software-defined network (SDN) [5] and network function virtualization (NFV) [6] have been revolutionizing telecommunications network design, including the fifth generation of mobile communications (5G) [7,8]. SDN improves network programmability [9], enabling slicing of the physical network devices. NFV supports virtualization of network functions that typically are implemented in hardware. Placement and chaining of virtual network functions (VNFs) create unprecedented levels of architectural flexibility. Multi-access edge computing (MEC) [10], fog and cloud computing can be combined to address VNFs optimal placement. By combining VNFs orchestration with SDN controllability, 5G architectures reflect service and application needs directly in physical device configurations, creating a disruption in the way that physical resources are managed. The work in 5G has been largely inspired/guided by previous work in future Internet (FI) research [11,12]. Future Internet means any Internet-like network that could emerge in the future. For instance, the outputs of FI public-private partnership (PPP) [13] have been fostering the 5G-PPP [14] initiative in Europe.

Disruption is also present in content distribution: information-centric networking (ICN) [15,16,17,18,19] replaces traditional host centric paradigm with efficient, coherent, secure and integral distribution of named contents. Packets identify the desired content instead of destination hosts. What matters are content names rather than locations. Services directly access contents/data by their names. Expressiveness is increased with named-content forwarding/routing [16]. Network caching can also be considered another disruption in this scope. ICN application to IoT management and control is a promising approach [20], since ICN provides in-network storage of control/management data. Access to the closest copy of a command is possible, as well as asynchronous interaction between controllers and controlled devices. Self-verifying names (SVNes) [21] can be applied to control plane, allowing provenance [22] and integrity check [23] in multi-controller networks.

The application of these disruptive technologies in smart environments will inevitably squeeze into the limited radio frequency spectrum of unlicensed bands, while providing devices, things and people connectivity. According to Haykin [24], the current static spectrum management policies are responsible for its poor use, leaving few bands for unlicensed use, challenging the coexistence of heterogeneous technologies in smart environments. Cognitive radio (CR) [25] can considerably increase the efficiency of radio frequency spectrum by performing a dynamic spectrum management (DSM) [24]. The dynamic assignment of spectrum holes (or free frequency channels) in a fair and efficient manner is a powerful tool to deal with congested frequency bands, reducing interference and improving throughput. Therefore, integrated, trustable and secure DSM for IoT, Wi-Fi and other technologies for unlicensed bands is extremely important for the current and future telecommunications networks. Technological solutions for this context must take advantage of the disruptive technologies mentioned above. To the best of our knowledge, the literature still does not present a unified combination of ICN, SOA, SDN, ID/Loc splitting and cognitive radio in favor to improve DSM on IoT environments [16,17,18,19,20,23,26,27,28,29,30,31,32].

In this article, we explore the benefits of integrating these disruptive technologies for DSM of WSANs and IoT networks. We advance state-of-the-art by leveraging: (i) named-based routing of control data to provide provenance and location-independent access to DSM controls; (ii) temporary storage of control data for efficient and cohesive control dissemination and asynchronous communication between software-controllers and controlled devices; (iii) contract-based control plane to improve trust-ability of control actions; (iv) service-defined configuration of wireless devices, approximating devices configurations to real services needs. These features bring fresh air to control plane, confronting the problems of confidence lack, security, reliability, consistency, provenance and integrity of control actions in WSANs and IoT networks [23].

Our approach for co-existence of wireless networks in the 2.4 GHz ISM band adopts a convergent design space (presented in Table 1) that integrates IoT, ICN, FI and 5G ingredients. The work is performed in the context of a FI/5G disruptive architecture called NovaGenesis (NG) [3,4,33]. NG project started in 2008 and includes support for several architectural ingredients, typically addressed in a standalone fashion. NG provides a complete architecture that covers ICN [15,16,17], SDN [5], service-oriented architecture (SOA) [34], service-centric networking (SCN) [35], NFV [6,31], self-verifying naming [3,16,21], distributed name resolution and identifier/locator (ID/Loc) splitting [36].

The article represents an extension of our previous work on cognitive radio for IoT [33], adding Wi-Fi and IEEE 802.15.4 channel control based on energy detection. A proof-of-concept has been experimentally performed, demonstrating throughput improvement after channel changing in a mobile environment, in which Wi-Fi interferes in IEEE 802.15.4 and vice-versa. A contract-based, named-data and software-defined approach is employed for IoT/Wi-Fi coexistence in the 2.4 GHz ISM band for the first time. The focus is on the control plane rather than previous work [33] that was mainly interested in ICN for data plane. To the best of our knowledge, this is the first effort for applying ICN in the control plane. In summary, the article objective is threefold: (i) to advance DSM for the co-existence of IoT IEEE 802.15.4 nodes and Wi-Fi access points in an ISM band; (ii) demonstration of NovaGenesis viability in a real scenario for integrated IoT/Wi-Fi spectrum management of smart places; (iii) demonstration of NG as an alternative to the current spectrum management architectures. Our main contributions are the following:

- Integration of aforementioned key ingredients usually separately found in IoT/FI/5G architectures. As it will be demonstrated latter, this work goes deeply than our previous work [33] when integrating the design dimensions, as reported in Table 1.
- Name-based routing and self-verifying naming for provenance and integrity of the best channel indications [23]. For the first time, name-based routing of the best channel indications is demonstrated in laboratory. In contrast to other works [20,22,26,27,37,38,39], results have been obtained in a field-trial experiments.
- “Semantic rich” orchestration of DSM services. NG provides naming support to foster trustable exposition and discovery of spectrum sensing, optimization and reconfiguration services. Dynamic composition is provided via publish/subscribe of name bindings.
- Configuration of IoT/Wi-Fi devices accordingly to the service contracts. IoT and Wi-Fi devices operate at channels defined by the umbrella of services contracts. This is more generic than only configuring traffic flow tables as in traditional OpenFlow-based SDN [5]. In the current SDN approaches, neither controllers are seen as services, nor contracts are established with services to reflect their real needs beyond traffic flow configuration. We propose a service-defined architecture (SDA), in which services and controllers establish dynamic contracts in the control plane, making devices configuration a direct reflex of services needs. In other words, our SDN model allows services to directly contract controllers to change physical device configurations.
- Network caching of spectrum data (at control plane) to improve scalability and efficiency of best channel indications for services. This solution allows asynchronous access to spectrum data, as well as trustable sharing of spectrum indications among DSM services. This solution is promising for scenarios with multiple spectrum sensors/best channel indicators and devices to be controlled.

In summary, this work aims at providing significant bit rate increase in IEEE 802.15.4 and Wi-Fi networks operating in unlicensed bands. The ICN-based proposal offers a spectrum management solution for IoT/Wi-Fi, using a named data network to switch from the current TCP/IP technologies to future Internet ones. The remainder of this manuscript is structured in other six sections. Section 2 presents a revision on the state-of-the-art in Section. In Section 3, we propose a cognitive radio system for the integrated spectrum sensing in the 2.4 GHz band, including its hardware and firmware modules. This cognitive radio system is latter integrated to NovaGenesis architecture. Section 4 describes the basics of NovaGenesis architecture. We also detail our main contribution: an extension to our previous work [33] to offer a set of trustable, named-control-data and contract-based dynamic spectrum management services for IoT/Wi-Fi devices. In Section 5, a real scenario is evaluated and experimental results are analyzed. Section 6 enumerates our proposal contributions, benefits and open issues for the control plane of new generation WSANs and IoT. Finally, Section 7 points our conclusions.

## 2. Related Work

Previous works related to the D1-D8 design dimensions are listed in Table 2. We have restricted our selection to the state-of-the-art manuscripts related to next generation technologies of WSANs and IoT. The hot topics and keywords presented in Table 1 have been used to select the best fits. As can be seen in Table 2, the majority of selected papers is typically focused on one or two dimensions. For instance, articles that cover cognitive radio-based DSM (D1 and D2) do not cover emerging technologies like ICN (D3 and D6), SDN (D4), SOA (D5 and D8), or identifier/locator splitting (D7). In this context, our methodology was selecting papers that cover as many as possible of these design dimensions.

The emergence of IoT and its perspective of providing connectivity for a very large quantity of devices [63] makes device/standard heterogeneity an important rule. Interoperability has been provided at software level. New technology coexistence problems have emerged and become largely studied. They include electromagnetic interference mitigation, dynamic spectrum management and opportunistic spectrum access.

The authors of [39,40,41,47,51,56] propose cognitive radio implementations for IoT, addressing channels keying with different techniques; not always related to the cognitive sensing technique, but employing multiple access protocol, multi-hop and others. In [40], cognitive radios were employed for IoT-enabled smart grids. The convergence of spectrum-aware communications and energy harvesting is explored to: (i) deal with collisions and interference in ISM band; (ii) rectify RF signals to feed IoT nodes. A ultra-low power unit perform channel sensing and switching in the nodes. In [47], cognitive radio technology is applied for vehicular communication in a licensed band. Opportunistic channels are allocated to vehicles as they move. The coexistence of medical wireless devices was investigated in [51]. A framework for wireless devices coexistence testing based on energy detection was proposed. Ding et al. [52] evaluated a distributed joint channel allocation and time slot access control solution for IoT devices, concerned to optimizing residual nodes energy. In [46], the coexistence of Wi-Fi and 4G in unlicensed spectrum was studied. Co-channel interference mitigation from small cells to Wi-Fi is provided, whereas in in [50] coordination of heterogeneous wireless sensor network technologies (Wi-Fi and IEEE 802.15.4) was investigated. The proposed WiSHFUL control framework addresses interference mitigation (D1) using an protocol-independent unified programming interface (UPI) for software-defined devices monitoring and configuration (D4).

In [55], authors reported an experimental performance evaluation of IEEE 802.11 and IEEE 802.15.4 standards, when sharing the same geographical space. In [26,38], SDRs were employed to have a more flexible network with quality of service (QoS). References [38,53,54,64] are related to SOA. Services designed to control the system operation and other functionalities aim to improve its performance, like the spectrum sensing service proposed in [53]. In [53], IoT devices with cognitive radio capabilities (D1) cooperatively optimize spectrum channels. Simulation and numerical analyses were employed to demonstrate minimal interference with licensed mobile networks. In [39], cognitive radio is employed for wireless sensor network energy-efficient channel handoff.

In [18], Jacobson et al. proposed content-centric networking (CCN) paradigm covering dimensions D3, D6 and D7. In CCN, pending interest tables (PITs) handle interest packets forwarded upstream to possible content sources in order to route back data packets to requesters. Zhang et al. [30] revisited CCN and proposed an enhanced version called named data networking (NDN). NDN also follows the same forwarding model, but proposes some improvements regarding delay. The surveys [19,29] cover many proposals also related to D3, D6 and D7. Network of information (NetInf) is one example [17] of a key component of the scalable and adaptive Internet solutions (SAIL) architecture. NetInf relays in named data objects (NDOs) to give access to content by its name, i.e., data is located during final transfer phase. Network caching is employed to store NDOs closely to the interested peers, maximizing content distribution efficiency and robustness. The NetInf nodes offer three services: (i) forwarding of requests for NODs to the closest caching/storage nodes; (ii) data transfer to the requesters; (iii) a name resolution service (NRS) that can be used to locate possible content sources. NetInf covers publishing, searching and subscription for NDOs. NetInf covers design dimensions D3, D6 and D7, clearly demonstrating there is a gap related to the other design dimensions while applying ICN paradigm. Still related to FI and ICN, the publish subscribe Internet technology (PURSUIT) architecture employed a flat naming structure (D6) to support content subscriptions from a publisher/subscriber rendezvous function at network nodes [32]. Publisher mobility is supported by a topology manager that decouples IDs from locators (D7). PURSUIT can support the secure exchange of control data, but there is no support for the dynamic composition of trustable services. Katsaros et al. [65] studied data-oriented architecture (DONA) [66] and context-ubiquitous resolution and delivery infrastructure for next generation services (CURLING) [67] route-by-name scalability and performance for hierarchical networking caching. The effects of inter-domain topologies in ICN performance have been evaluated, pointing this issue as important to be considered when extending ICN for hierarchical domains. Another important aspect related to ICN is proactive caching of contents accordingly to user mobility. Siris et al. [68] proposed an efficient mechanism to reduce delay when mobile devices employ ICN paradigm, which is an important problem for the convergence of ICN, IoT, 5G and cognitive radio. The proposed solution exploits mobility and congestion information to improve cache storage performance.

Additionally, we selected studies that tackles ICN benefits for IoT. The authors of [20,23,26,27,28,42,43,44] addressed the application of ICNs to IoT. In [20], content-centric network (CCN) was employed for management of IoT networks. Name resolution (D6) and name-based, secure routing (D3) was used for IoT devices key distribution, registration, discovery and command execution (D2 and D4). They have also considered service level agreement (SLA) for management services (D8). CCN offers content identifier/locator splitting (D8). In contrast, our work employs ICN for wireless spectrum management (D1). Another difference is that we adopt service names for dynamic composition of IoT/Wi-Fi spectrum management services (D5).

In [22], motes authentication (D2) and IoT data name-based routing (D3) were explored in the context of the named-data network (NDN) architecture. Scalability and efficiency were evaluated using ns-3 simulations. In [27], a simulation-based comparison of Internet protocol (IP) and CCN (D3 and D6) for supporting IoT data plane was provided in terms of energy consumption and bandwidth usage. Results indicated CCN consumes less energy and bandwidth than IP. In [28], two ICNs were evaluated in the context of IoT: MobilityFirst [69] and NDN. The study covered devices discovery and IoT data publish/subscribe. Two smart campus applications were evaluated with simulation.

In [42], an ICN-based middleware for IoT was proposed. The proposal covered IoT devices naming, exposition and discovery. Name-based IoT data dissemination was evaluated in a real scenario. Nour et al. [43] enhanced NDN architecture towards devices naming (D6), management (D2), mobility (D7) and handoff. Simulation results contended on proposal’s scalability and efficiency. Another paper related to ICN name and name resolution in IoT context was proposed by Dong et al. [44], which covered devices attachment and failures. In [23], security of ICN-IoT (D2, D3 and D6) was investigated. The work is related to the ICN research group (ICNRG) of Internet research task force (IRTF), which investigate ICN application in the context of IoT. An ICN-IoT middleware was proposed to provide service (D5 - partially), device and content (D3) discovery. A secure naming service (D6) was also covered. In [48], authors extended NDN to reduce IoT data latency. Adhatarao et al. [58], employed CCN-Lite for IoT nodes and gateways. The work addressed naming in IoT, publish/subscribe communication model, security and mobility.

The convergence of ICN (D3 and D6) and CR (D1) has been explored in a very few works. Si et al. [26] addressed efficient video distribution in this context. Mathematical modeling and simulation were employed. Although the concern was to improve spectrum efficiency, the work has no relation to D4.

Another convergence is spectrum management (D1) and SDN (D4). In [37], an enhanced distributed channel access (EDCA) technique was integrated to an SDN controller. Access points and mobile devices configured channels accordingly to a SDN controller. CORAL-SDN [49] was embedded (D4) inside IoT nodes to offer low power wireless communication and media access control in IEEE 802.15.4 2.4 GHz networks. A modified controller offered topology control, routing and flow establishment, and data collection. Spectrum management was left out of the scope. In [9], Lin et al. proposed a programmable P4 switch that provided aggregation/disaggregation of IoT data.

The integration of SDN and NFV is relevant for convergent architectures. Although independent, the synergistic integration of SDN and NFV can take advantage of both disruptions simultaneously. Duan et al. [59] proposed an integrative approach called software-defined network virtualization (SDNV). This approach provided not only separation of data and control planes, but also decoupling of service functions and infrastructure. A service-oriented control/management (D2, D4 and D5) plane was provided to allow independent coordination of physical (infrastructure) and virtual (service-based) resources. Duan et al. [59] contended that unified abstractions are required to expose heterogeneous resources to software layer, e.g. information processing, networking and storage. However, the SDNV proposal did not covered this issue. Coherence of coexisting software-controllers (control plane) is still pointed as an open issue. Service-oriented proposals (D5) are also relevant for next generation IoT and smart places. They are typically related to cloud or fog computing. In [45], a service-oriented platform for IoT devices integration and behavior-aware coordination was proposed. The work employed the devices profile for web services (DPWS) standard to support heterogeneous IoT devices, including IEEE 802.15.4. Discovery and control services were developed (D5), but none related to coexistence of RF signals. In [57], a SOA-based middleware for wireless sensor networks addressed services security (D2 and D4), dynamic composition (D5), data aggregation and QoS challenges of smart environments. In [62], a data oriented networking architecture (DONA) based on cloud computing was proposed. It addresses cloud orchestration for ICN, covering the challenges behind IoT and its control plane.

A novel naming structure for identifying and locating IoT data and services (D7) is discussed in [61]. Different rules for controlling information distribution are proposed and organized into hierarchical caches. A user that desires to obtain an information asks for the scope corresponding to a certain context, i.e., it can access IoT information by their names. In [60], an example of hierarchical network caching (HNC) is provided, as well as a mechanism for mobility-based data search. These works address the important issue of ICN-based hierarchical caching performance for IoT.

Regarding future Internet, the eXpressive Internet architecture (XIA) is a FI project funded by the USA national science foundation (NSF) [16]. Its key concepts are: (i) employs global unique identifiers for networking principals, i.e., administrative domains, hosts, services, and contents; (ii) allows services to express intent via unique IDs; (iii) flexible addressing; (iv) iterative refinement of forwarding; (v) intrinsic security. Self-certifying identifiers (XIDs) are calculated by hashing the public cryptographic keys of domains, hosts and services or the entire binary pattern of named-data. Still related to FI, recursive Internetwork architecture (RINA) is a clean slate architecture founded on the interprocess communication (IPC) abstraction [31]. RINA adopted a distributed IPC facility (DIF) to allow dynamic stack creation based on customized instances of recursive layers. In other words, DIFs have the same interface and structure, independently of their level in the protocol stack. IPCs can implement virtual network functions. RINA also has an inter-DIF directory (IDD) for name resolution (D3 and D6) and ID/Loc splitting (D7). An error and flow control protocol (EFCP) provides secure exchange of control data (D2). A common application connection establishment phase (CACEP) is employed for establishing application connections (D8), allowing applications to discover (D5) possible peer names. To the best of our knowledge, neither XIA, nor RINA have been applied for cognitive radio and DSM yet.

In summary, related work individually deal with the challenges of next generation IoT and wireless network demands, providing mathematical models and/or simulations, with a few real world implementations, addressing specific design dimensions of Table 1. The majority of cognitive radio proposals do not address other design dimensions. This means they do not form trustable networks of control services, if possible implications in security. Moreover, they do not exchange control data by their names, lacking on provenance and integrity check of control actions [22]. Name-based routing is also unavailable, making control actions dependent on node’s addresses and locations. Control plane is not software-defined, lacking in flexibility and evolvability. Control services are not dynamically composed, difficulting extensibility and requiring frequently manual intervention. Naming support is limited to the current Internet technologies, difficulting expressiveness [16,45]. Devices, services and data identifiers are coupled with locators, which creates identity loss and traceability problems while moving. Control plane is not contract-based, meaning all control services do not follow pre-established quality requirements. ICN proposals address some of these limitations, but do not incorporate cognitive radio (or DSM) or SOA benefits. Suarez et al. [20], applied ICN for IoT management, but have not covered cognitive radio or SOA. In summary, there is a gap in the current researches to fully address design dimensions listed in Table 1 to automatic switch wireless devices’ channels (not only for Wi-Fi, but also for IEEE 802.15.4) using ICN, SOA, SDN and cognitive radio, while optimizing network throughput.

## 3. Cognitive Radio System

This section contributes with a protocol-agnostic ISM band cognitive radio system to dynamically determine the best channels in the Wi-Fi/IEEE 802.15.4 networks. It measures energy level for either IEEE 802.11 and IEEE 802.15.4 over the 2.4 GHz frequency band. Its objective is to increase throughput when IoT and legacy wireless network coexist. This CR system can interoperate with or without NovaGenesis architecture, as will be described in Section 4, Section 5 and Section 6, respectively. This dual approach enables us to compare both solutions, contrasting the novelties behind NovaGenesis design.

### 3.1. Hardware

**Figure 1:**
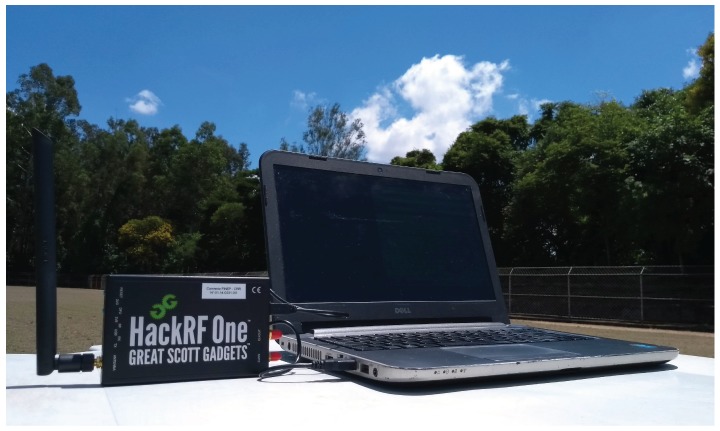
Hardware used to run the sensing cell module and the channel advisor (CA) firmware.

The implemented system is composed by a sensing cell module and a wireless device network. Figure 1 shows the sensing cell hardware constitution, which uses an open source software defined radio, called HackRF One™ to sense the frequency spectrum and a laptop with middleware to process signals and perform SDR control. The HackRF One operates from 1 MHz to 6 GHz and has 20 MHz bandwidth, which may be controlled through a middleware/software in any hardware with more at least 1 GHz of processing clock. Our laptop has an Intel(R) Core™ i7 processor, with a 1.80 GHz clock rate. The IoT network considered in this experiment operates in the IEEE 802.15.4 communication standard. To evaluate the interference caused by other wireless devices, which usually coexist with IoT nodes, an IEEE 802.11 network is additionally deployed. The IoT 802.15.4 network is constituted by a SMARTRF06EBK as the network border router and Texas Instruments cc2650 motes. The Wi-Fi network uses a TP-Link N750 OpenWRT router and two laptops communicating via TCP/IP socket to create data plane traffic. In other words, our novel approach operates in the control plane, optimizing coexistence of traditional technologies for IoT.

### 3.2. Firmware

For the sake of clarity, we divided firmware component in two parts: (i) the algorithm for the best channel estimation; (ii) the employed spectrum sensing method.

#### 3.2.1. The Best Channel Estimation Algorithm

The main script of the spectrum sensing system, named as Channel Advisor, which starts with the indication of the link layer technology to be sensoried (Wi-Fi or IEEE 802.15.4) coming from NovaGenesis, when it is employed or automatically (without NG benefits) when it is not present. The message is received through a zero-em-queue (ZMQ) socket (TCP/IP) and indicates whether the spectrum sensing service should be ON/OFF; when it is ON, the wireless communication standard being sensoried. As it will be described latter in Section 4.9.1, a few characters (“ON 802.15.4”) are send by NovaGenesis spectrum sensing service (SSS) to turn ON channel indications and inform the frequency bands of the technology to be scanned.

**Figure 2:**
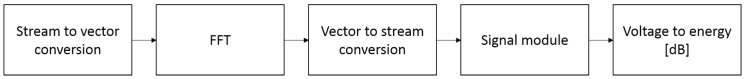
The GNU Radio sensing algorithm flowchart.

Figure 2 describes the employed GNU Radio sensing algorithm flowchart. Once the main script receives the first NG message, it initiates the firmware to control the SDR. The firmware interacts with a GNU Radio instance, which is a set of open source tools for SDR implementations. We developed a GNU Radio algorithm (implemented in a set of communication blocks) that transform the sensed data stream into a 1024 positions vector. In the next step, the vector is converted to the frequency domain through a FFT block. Later, the module of the generated data stream is calculated and converted from the voltage scale to the energy scale.

**Figure 3:**
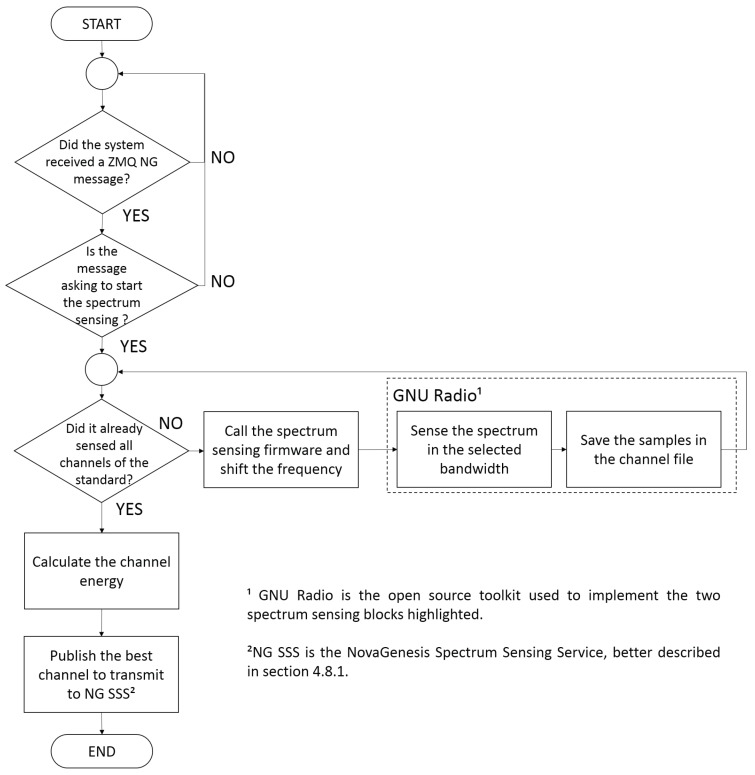
The best channel advisor complete flowchart.

Figure 3 illustrates the flowchart of the Channel Advisor firmware. It is important to mention since the HackRF One has a bandwidth of 20 MHz and the 802.11 channels have 22 MHz, the GNU Radio sensing flowchart is repeated two times for each 802.11 channel. Therefore, the script can sense 11 MHz each time and all the channels are covered. Sensing the 802.15.4 standard is not a problem, since its channels have a 5 MHz bandwidth. The energy samples of each channel are stored into binary files. After sensing all channels, the main script calculates the channel energy and chooses the channel with less energy collected from other coexisting devices. Posterior, the Channel Advisor inform the best channel to NovaGenesis, as will be described in Section 4.

#### 3.2.2. Spectrum Sensing Method

In cognitive radio, a typical hypothesis test represents the spectrum sensing [70]:

(1) $$\left\{\begin{array}{c} H_{0}:\;\mathrm{Primary}\;\mathrm{signal}\;\mathrm{is}\;\mathrm{absent}\\ H_{1}:\;\mathrm{Primary}\;\mathrm{signal}\;\mathrm{is}\;\mathrm{present}, \end{array}\right.$$

where $H_{0}$ denotes there is no primary user in a particular frequency band, whereas $H_{1}$ represents that a specific band is occupied by a licensed user.

One can measure the spectrum sensing performance through the probability of detection, $P_{D}$, and probability of false alarm; $P_{FA}$. $P_{D}$ and $P_{FA}$ are described as [70]:

(2) $$\begin{array}{c} P_{D}:\;Pr\left(H_{1}|H_{1}\right)=Pr\left(T>\gamma |H_{1}\right)\\ P_{FA}:\;Pr\left(H_{1}|H_{0}\right)=Pr\left(T>\gamma |H_{0}\right), \end{array}$$

where Pr is the probability of an event; T is the test statistic of the spectrum sensing method and γ is the decision threshold. When T is above γ, the channel is taken as occupied, otherwise the channel is assumed to be free. Each spectrum sensing technique in a cognitive radio is able to calculate the test statistic T, as demonstrated by Equation (2).

There are many techniques to set the test statistic T, including the matched filter, cyclostationary, energy detection and others. Considering the known methods, the energy detection is the only approach that does not demand total or partial knowledge of the signals to be detected, not even about the communication channel [71]. This condition is fundamental to sense an IoT devices scenario with different and unknown standards. For this reason, the energy detection method has been chosen as the spectrum sensing technique to be implemented in our system.

In the energy detection method, the test statistic for N received samples is calculated by [72]:

(3) $$T=\frac{1}{N}\sum_{n=1}^{N}\left|X{\left(n\right)}^{2}\right|$$

The sum of the measured samples potency module, according to 3, is performed as illustrated in Figure 2. Since the noise in a real environment is dynamic, estimation errors could lower the technique performance if the threshold decision γ is wrong. Besides that, with the huge amount of IoT connected devices, it becomes possible that in a certain moment all sensed standard channels are occupied. This work evaluates the benefits in a scenario of less interfered channel of a certain standard (Wi-Fi or IEEE 802.15.4). Therefore, a threshold is not calculated, but the channel with less measured energy is determined. This criterion is used for the execution of the flowchart as shown in Figure 3. Once the system establishes the channel with less measured energy, it switches the network to the operation at the new channel and the process can be repeated every time other channel offers less interference, respecting the minimal difference calibrated to cause the exchange.

## 4. NovaGenesis

NovaGenesis (NG) [3,33] is an alternative information architecture that covers data processing, exchanging and storage. The project has idealized an alternative architecture for the current Internet and has been demonstrated viable for IoT [4] and cognitive radio [33]. NG has been evaluated as an alternative to domain name service (DNS) [3], named-data routing and network caching [73] (for efficient content distribution) and software-defined networks [74]. Due to its service-oriented design (SOD) with support for virtualization, software-defined networking and virtual network functions, it can be seen as a 5G architecture as well. In this context, NovaGenesis is a “clean slate” architecture that cohesively integrates many novel ingredients at its core, including exposition of physical things to software, programmable networks (SDN and NFV), service-defined architecture (SDA), ICN, SOA, SCN, IoT, SON, among others.

### 4.1. Naming and Hierarchical Name Resolution

NovaGenesis allows for unlimited namespaces and provides distributed/hierarchical name binding resolution. Namespaces can cover from natural language names (NLNes) up to self-verifying names (SVNes). For instance, a SVN can be calculated from an IoT hardware serial number, i.e., SVN = MD5(12080180972B) = b09d3a2e3aead0308403ecb0d6c6f9a4, in which MD5 is a hash function. Moreover, NG supports devices NLNes, such as NLN = Mote 1, which are important for human operators.

Besides flexible naming, NG allows name bindings (NBs) to represent entities relationships by means of semantic operators, such “equivalent”, “is contained” or “contains”. For instance, a name bind < Mote 1, b09d3a2e3aead0308403ecb0d6c6f9a4 > stores that one existence have two equivalent names, i.e., homonyms. However, a “contains” NB can represent that some entity inhabits another one. For example, the NB < Domain 1, Mote 1> represents that the domain named Domain 1 contains a Mote 1. NG allows also name bindings to be associated with the contents, i.e., data objects that are distributedly stored in the network. In this case, a name “Sample1.json” can be associated with a binary file containing a certain measure.

### 4.2. Hierarchical Network Caching

NovaGenesis adopts temporary caches in every domain to improve named-content distribution. This approach is founded in ICN, in which contents are accessed by their names and popular information is made available to future access in local cache. NG name resolution approach is integrated to a network caching to form a name resolution and network caching service (NRNCS). By integrating name resolution to content caching, NG facilitates content delivery by their names.

The current implementation of NRNCS adopts a publish/subscribe communication model, in which services publish and/or subscribe name bindings (NBs) and associated contents (if any) from the local domain cache. Therefore, NRNCS provides a rendezvous point analogous to message queuing telemetry transport (MQTT) brokers.

NRNCS is implemented by three services: (i) publish/subscribe service (PSS) to which services can publish or subscribe NBs/contents; (ii) generic indirection resolution service (GIRS) that selects appropriate hash tables to store named data; (iii) a hash table service (HTS), which in fact stores NBs and associated contents. Every NG domain must have at least one instance of each of these core services. However, elasticity is provided by increasing the numbers of PSS/GIRS/HTS. To facilitate understanding, a summary of NG terminology is shown on Table 3.

PSS forwards NG messages carrying name bindings and content for network caching. For this purpose, it exposes an application programming interface (API), which offers the following primitives: (i) NB and content publishing without notification of other services; (ii) NB and content publishing with notification of interested services; (iii) NB and content subscription; (iv) NB and content subscription with notification of publisher; (v) delivering of NB and content subscribed; (vi) revoking of published NB and content, if any. In summary, PSS offers an API distributed over the network, allowing services discovery and access based on naming.

### 4.3. Entities Life-Cycling: From Equipment, Operating Systems, Services up to Information

NG adopts SOA premises to perform everything-as-a-service (XaaS) dynamic composition. Event protocol implementations in software are seen as services that establish contract to other services (peer protocol implementations or applications). NG provides a rich service life-cycle with resources (capabilities) exposition, discovery of possible peers and contents, contracting offers, negotiation and SLA installation. Due to its unique naming and name resolution approach, NG enables name-based dynamic service composition. In other words, NG integrates service-centric networking (SCN) and ICN in an unique design. Not only access to information is name-based, but also access to services. In fact, access to all entities is name-based as well as content routing and delivering. All these NG features provide a service-defined, trustable IoT spectrum management approach.

### 4.4. Message Encapsulation over Link Layer

Although NG can be applied to link layer, the current implementation does not cover this feature. As a consequence, NovaGenesis messages must be adapted for contemporary link layer technologies, such as Ethernet, Wi-Fi, ZigBee, LoRa, etc. For this aim, a gateway service was designed. In the current implementation, a convergent proxy/gateway/controller service (PGCS) was developed to represent (as a proxy) ordinary things (and IoT nodes) inside NG service ecosystem. PGCS provides gateway (protocol translation) functions to IoT protocols, such as ZigBee, IEEE 802.15.4, LoRa. Additionally to message encapsulation, NG model requires a service to represent things at software layer. Currently, this idea is named smart object. For this purpose, PGCS includes an internal proxy component which represents things in NG life-cycle.

### 4.5. Layered Model

**Figure 4:**
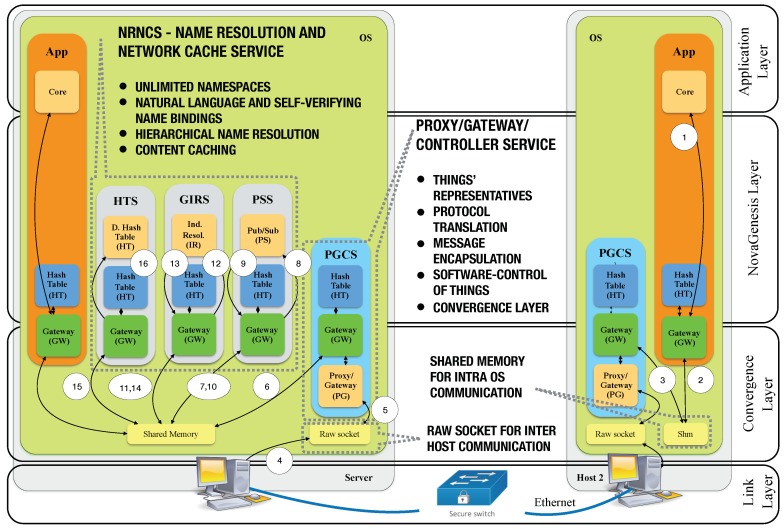
A layered model of the NovaGenesis architecture in a local domain. The convergence layer adapts NG messages to be transported in current link layer technologies. The NG layer comprehends core NG protocols that support application layer via a PSS application programming interface (API). NovaGenesis dynamic stack enables customization of the protocols required for a certain application. Therefore, overhead of unnecessary protocols can be avoided. The convergence layer provides message encapsulation, fragmentation and reassembly. The NG layer provides name-based message forwarding, content delivery and storage. We have already embedded a simplified version of this stack for IoT [4].

Even though NG allows for flexible protocol development and layering, the current implementation can be represented in a protocol stack as illustrated in Figure 4. The model presents two computers hosting NG. Each NovaGenesis service has several internal components to implement their functions. These components are called blocks, which can be classified in two types: common and specialized. The following blocks inhabit all services: gateway (GW) and hash table (HT). GW provides: (i) inter block communication (IBC) inside a service; (ii) inter service communication (ISC) in an operating system (OS); and (iii) event-driven dispatching of messages and callback of service actions. Inter OS/host communication is done by a specialized block called proxy/gateway (PG), which implements a convergence layer to provide message encapsulation, fragmentation and reassembly. PGCS, PSS, GIRS and HTS offer a software implementation of the NovaGenesis layer. Finally, in the upper level of the architecture an application layer takes advantage of all core services.

### 4.6. Software-Defined Networking Model

**Figure 5:**
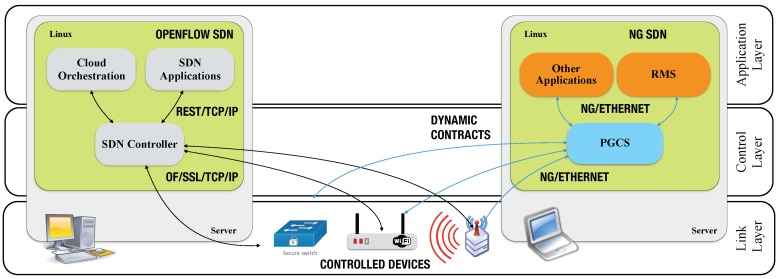
Comparison of OpenFlow-based SDN with NovaGenesis service-defined architecture (SDA).

PGCS can also control things, self-configuring them as required, based on dynamic established contracts (Figure 5). PGCS was envisioned as a controller service, similar to a SDN controller, but with responsibilities larger than frame forwarding control. PGCS provides programmability of connected things, sensors, actuators and switches. It can change general configurations in represented devices. In an OpenFlow-based SDN, software-controllers employ OF protocol to configure flow tables in compatible switches. OF protocol is employed in the south bound, between controller and switches. In the north bound, REST is typically employed, e.g., in OpenDaylight and open network operating system (ONOS). Figure 5 provides a comparison between OF and NovaGenesis SDN models.

NG does not limit the scope of programmability to flow tables. It employs services life-cycling to dynamically compose control plane via service contracts. NG applies the same protocols at north and south bounds. PGCS represents things to “sell” their capacities via NG named-based, semantic rich service orchestration. PGCS configures things accordingly to high level service and application needs, creating a service-define architecture (SDA) [4]. When applied for IoT spectrum management, PGCS can represent IoT nodes to sell their measurement capabilities to control applications, such as a RMS instance. In addition, it can change the RF channel being used by the IoT node to communicate. PGCS can also change flow tables in a switch or access points in a similar way to OpenFlow, but using NG publish/subscribe communication model. It can also interoperate with OF controllers [74].

### 4.7. Publishing Content to the Local Domain Temporary Cache

Figure 4 demonstrates the path required to publish a NG message from an application to the NRNCS. In Transaction 1, a Host 2 application publishes a message to the PSS. The local gateway determines the message is destined to another host (using a host identifier in the message header). The App GW forwards the message to the local PGCS GW via Transaction 2. The PGCS GW also determines the message destination is Host 1 and forwards the message to its PG for inter OS message exchange (Trans. 3). The PG block employs a raw socket to encapsulate NG over Ethernet (Trans. 4). Then, Host 1 PGCS PG reads the message from raw socket (Trans. 5). Using PSS identifier (also contained in message header), the PGCS GW forwards the message to the PSS instance (Trans. 6 and 7). The message goes to PS block, where a GIRS instance is selected to continue storage (Trans. 8-10). The message arrives at the GIRS GW and is forwarded to the IR block (Trans. 12). The IR block selects the proper HTS instance to store the content and forwards the message to HTS GW (Trans. 13–15). The message arrives at the HTS HT block (Trans. 16), where its content is store in Host 1 Linux file system. NRNCS always involve message forwarding from PSS to GIRS and from GIRS and HTS. A content subscription follows the same path up to the HTS. Content delivery is done by HTS directly to the subscriber service.

### 4.8. Message Format

NovaGenesis messages are divided into two portions: command lines (that call Actions in the receiving side) and payload. The command lines are in ASCII format, employing a novel script language to define control commands and their parameters. The payload is streamed from a file system archive. To separate command lines and payload a blank line is employed. The structure of each command line is as follows:

ng -command –alternative version [ < n type E1 E2 E3 E4 … En > ]

in which

-command identifies the Action that will be called at the destination.

-alternative identifies possible alternatives to the command line, allowing Actions customization.

version identifies the version of the command line being executed.

[ ] contains one or more vectorial arguments.

n indicates the number of elements in the vector.

type contains the type employed in the vector elements.

E1 E2 E3 E4 … En are the vectors’ elements, containing parameters for the command line.

**Figure 6:**
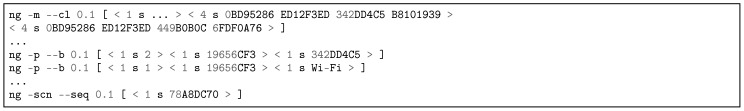
Example of a NG exposition message. In the current prototype, NG messages carry command lines with one or more arguments. The messages are textual, to facilitate development. Future versions will include source encoding to reduce overhead.

Figure 6 gives an example of a NovaGenesis exposition message used by a service to publish its name bindings to other services. The first command line with the command ng -m –cl is a forwarding/routing command line [75], which contains the control information required to forward the message to a certain destination. It contains two tuples of four values that identifiers respectively, source and destination self-verifying names (SVNes). The argument [ < 4 s 0BD95286 ED12F3ED 342DD4C5 B8101939 > ] contains a tuple that names the source of the message and the argument [ < 4 s 0BD95286 ED12F3ED 449B0B0C 6FDF0A76 > ] provides the names of the destination. The command line ng -p –b is used to publish name bindings, therefore exposing keywords related to the service exposing its features, e.g., the key 19656CF3 is the hash of the word “Wi-Fi”. The last command line contains the hash of the previous command lines for integrity check purpose.

### 4.9. Dynamic Spectrum Management with NovaGenesis

NovaGenesis allows spectrum sensing, management and control as a service. The service-oriented spectrum management components are implemented as services in NG environment. They represent spectrum sensing hardware to enable dynamic composition with a spectrum manager. Services also represent the Wi-Fi access points and IEEE 802.15.4 nodes, allowing software-defined configuration of wireless channels. NG enables contract-based spectrum management, integrating all components required for dynamic spectrum access (DAS). It aims to create a dynamic spectrum market, in which opportunistic usage is allowed and monetized accordingly to established contracts or SLAs.

#### 4.9.1. Spectrum Sensing Service (SSS)

**Figure 7:**
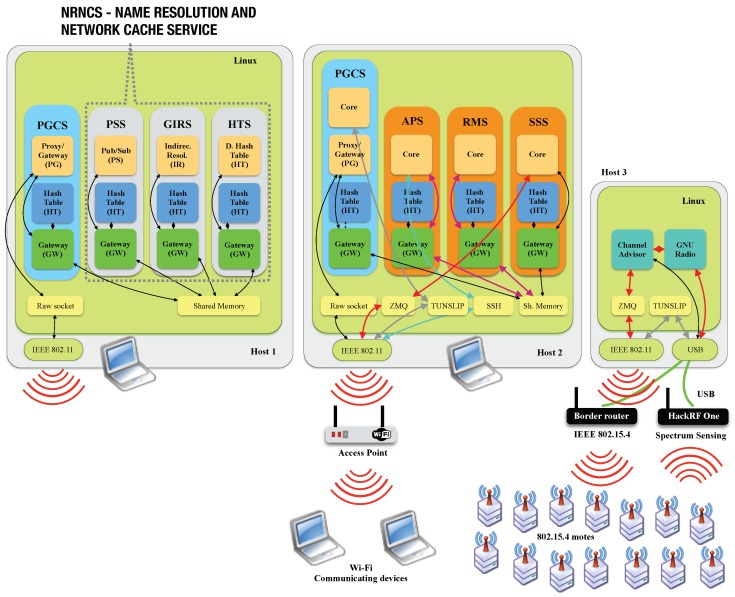
Joint IoT and Wi-Fi spectrum management with NovaGenesis. The Host 1 is running NG core services, while Host 2 is running NG spectrum management services, including spectrum sensing service (SSS), access point service (APS) and resource management service (RMS). SSS provides best channel indications to RMS based on HackRF One™ measures. RMS manages channel indications and selects best channels for Wi-Fi and IEEE 802.15.4 devices. APS configures the best channel to be used in Wi-Fi access points. Observe that PGCS at Host 2 has a core block, which implements service offering and acceptance from RMS. Host 3 is running Channel Advisor (CA) and GNU Radio.

In this paper, we extended a previous spectrum sensing service (SSS) development [33] to expose IEEE 802.11 and IEEE 802.15.4 best channel indications to a resource management service (RMS). As illustrated in Figure 7, measurements performed in HackRF One™ hardware are transferred to a GNU Radio instance. The channel advisor (CA) controls spectrum energy measurement and connects to a Core block of SSS via zero-em-queue (ZMQ) request/response socket. In addition, SSS also publishes keywords (“SSS”, “Spectrum”,“Sensing”, “IEEE 802.15.4” and “Wi-Fi”) to other NG spectrum management services in a local domain via PSS. Posterior, SSS searchers for a RMS instance. When SSS discovers a RMS, it sends a service offer to RMS describing the details behind best channel indications that will be performed.

#### 4.9.2. Access Point Service (APS)

APS is novel service to represent and control Wi-Fi access points inside NovaGenesis. APS publishes keywords (“APS”, “Access”, “Point”, “Controller” and “Wi-Fi”) to other NG spectrum management services to enable dynamic composition. It also searchers for RMS instances and sends a service offer to discovered ones, describing details of the Wi-Fi APs it is representing. A service acceptance message will be sent by the RMS in case of contract establishment. Then, APS can exchange channels in Wi-Fi APs as requested by RMS. For this aim, APS Core block sends a control message to the AP via SSH. APs need to run OpenWRT OS.

#### 4.9.3. Resource Management Service (RMS)

RMS implements a name-based, contract-oriented, self-organizing wireless spectrum management approach in a local NG domain. RMS mediates the relationship among NG spectrum management services (a.k.a SSS, APS and PGCS). Communication among them is TCP/IP independent. When a SSS discovers a RMS, it proposes a service offer with a description of the channel indication functionality that will be provided by CA software (Section 3). RMS accepts the contract and starts being notified of the SSSes’ publications which inform: (i) the wireless technology that is being sensed (Wi-Fi or IEEE 802.15.4); (ii) the best channel to be used by local devices. Therefore, RMS becomes aware of the radio spectrum situation (a property called situation awareness) in the industrial, scientific and medical (ISM) bands. RMS can then decide if it accepts latest indication subscribed from SSS or to keep the current configuration. If it decides to change Wi-Fi/802.15.4 channel configurations, it publishes a control message to one or more local domain APS/PGCS, asking them to change channel. PGCS is used in case of IEEE 802.15.4.

#### 4.9.4. Proxy/Gateway/Controller Service (PGCS)

When an IoT node needs to change the frequency channel, e.g., a IEEE 802.15.4 node, a contract to its representative PGCS is required. In this way, PGCS exposes its keywords and sends a service offer to RMS. The offer exposes PGCS IoT node control feature, which can be done via Tunslip software as illustrated in Figure 7. Future work will enable IEEE 802.15.4 channel exchange without using TCP/IP. An embedded proxy/gateway service (EPGS) is being adapted [3] to run inside IoT nodes and accept NovaGenesis messages directly, without TCP/IP.

### 4.10. Dynamic Composition of Spectrum Management Services

**Figure 8:**
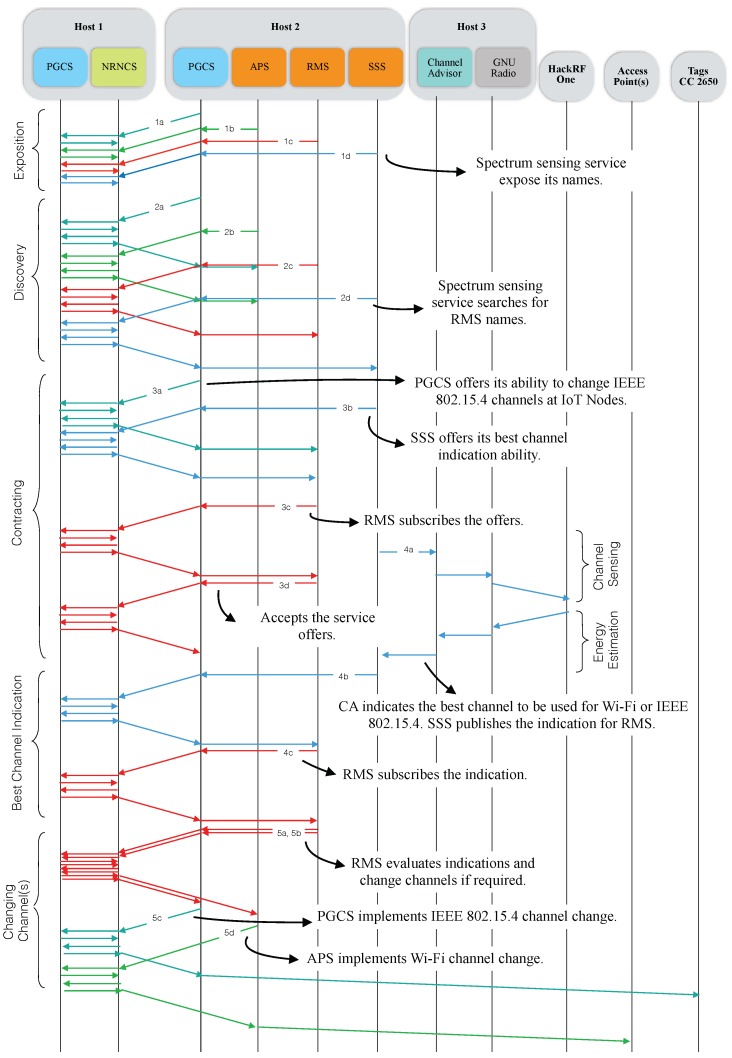
Life-cycle of the dynamic spectrum management approach with NovaGenesis. Five steps are shown: service exposition, service discovery, contracting (dynamic composition), best channel indication and channel changing (Wi-Fi and IEEE 802.15.4). Each vertical line represents the control plane actions related to a certain component. Control actions (Transactions) are named as nc, in which *n* is related to the life cycle step being performed and *c* is a sequence number introduced to facilitate understanding. For instance, 1a is a control plane message send by PGCS to NRNCS.

Figure 8 provides a sequence diagram of the actions implemented to dynamically manage 2.4 GHz ISM spectrum with NovaGenesis. The life-cycle comprehends five steps: (i) service names/keywords exposition; (ii) service discovery; (iii) service contracting; (iv) best channel indications; (v) device channel adjustment.

#### 4.10.1. Service Names/Keywords Exposition

In Transaction 1a, PGCS from Host 2 publishes to NRNCS a set of self-verifying name bindings, e.g., among Host ID, OS ID, Process ID and internal component IDs. In addition, SVNes are bound to natural language names (keywords) to express PGCS role in spectrum management solution (e.g., “Proxy”, “Gateway”, “Controller” and “IoT”). These NBs are stored in the HTS distributed system. Transactions 1b, 1c and 1d carry, respectively, name bindings of APS, RMS and SSS instances. SSS keywords are “Spectrum”, “Sensing”, “Service”, “Wi-Fi” and “IoT”, while RMS are “Manager”, “IoT”, “RMS” and “Management”. Other keywords can be used, in any language. These transactions are forwarded by local Host 2’s PGCS to the Host 1’s PGCS, in which NRNCS is running. In the end of this exposition phase, all name binding (graph of names) required to self-organize spectrum management services are stored in the NRNCS.

#### 4.10.2. Service Discovery

In the service discovery step, services subscribe NBs can identify possible peer services for the solution. In Transaction 2a, PGCS subscribes the keywords “Manager”, “IoT”, “RMS” and “Management” in order to discover a RMS instance. APS and SSS also employ the same keywords, while searching for RMS (Transactions 2b and 2d). In the opposite direction (2c), RMS tries to discover PGCS, APS and SSS instances that can be employed to control devices. NRNCS delivers NBs resulting from these searches to each one of the querying services. This service discovery procedure is distributed, periodic and recursive. Not all the Transaction taken are shown in Figure 8 for the sake of simplicity.

#### 4.10.3. Service Contracting

In Figure 8, two service contracting actions are illustrated. In the first one, PGCS publishes a service offer to a RMS instance that it discovers. In this offer (3a), PGCS informs its ability to exchange IEEE 802.15.4 channel at IoT sensor tags it represents. In the second one (3b), SSS offers to RMS the ability to indicate best channels to be used not only in Wi-Fi ISM band, but also in IEEE 802.15.4. In a third action (not shown in Figure 8), APS offers to RMS its ability to exchange Wi-Fi channels at access points. All offers are forwarded to RMS, which coordinates all actions in a domain.

RMS is notified by NRNCS about the three service offers commented above and subscribe them (3c). After receiving the service offers (in a .txt file), RMS analyzes its contents. In Transaction 3d, RMS accepts the service offer by publishing a service acceptance .txt file to NRNCS and notifying PGCS about it. PGCS subscribes this service acceptance object and stores it locally. The other service contracts are established in a similar way.

#### 4.10.4. Best Channel Indication

After establishing all contracts, services can perform dynamic spectrum management via named-content message exchanging. In Transaction 4a, SSS queries channel advisor software about the best channel to be used in a domain. Queries can be applied for Wi-Fi or IEEE 802.15.4. CA determines the best option based on a GNU Radio instance connected to a HackRF One™ software-defined radio. In Transaction 4b, SSS publishes a best channel indication to the local domain cache (NRNCS), which notifies RMS about this publication. RMS subscribes the best channel indication and decides if a channel exchange is necessary.

#### 4.10.5. Devices Channel Adjustment

The last step is to change devices channels according to the need. RMS can change one or more access point channels or IoT node channels. In the case of changing IEEE 802.15.4 channels, RMS publishes a channel change control message to the PGCS (5c). PGCS is notified about this publication and subscribes it, implementing a properly channel change in the IoT Sensor Tags. In the contrary (Wi-Fi channel changing), RMS publishes a control message to the local domain APS (5d). APS subscribes the control message and implements a channel change in the AP(s).

## 5. Experimental Results and Analysis

Our architecture can be employed to any kind of wireless communication protocol, since SDR senses all RF signals present over a desired frequency bandwidth. To evaluate the system efficiency for an IoT scenario, we choose two standards that might be commonly seen in the same ambient: (i) IEEE 802.15.4, composed by fourteen IoT motes sharing the geographical space; (ii) IEEE 802.11 (Wi-Fi) devices.

### 5.1. Testing Methodology

All measurements were executed in an open soccer field, apart from other 2.4 GHz interference signals not considered as part of the experiment. At data plane, the Wi-Fi network has been composed by one router (an access point or AP) and two Wi-Fi end devices. The AP is a TP-Link N750™ with OpenWRT, covering features like operation bandwidth control, potency control and channel management, all configured by software. The Wi-Fi end devices were a laptop and a mobile phone, both connected through a TCP socket generating traffic, continuously. The 802.15.4 network was composed by one border router Smart RF06™ and fourteen Texas Instruments cc2650™ motes.

**Figure 9:**
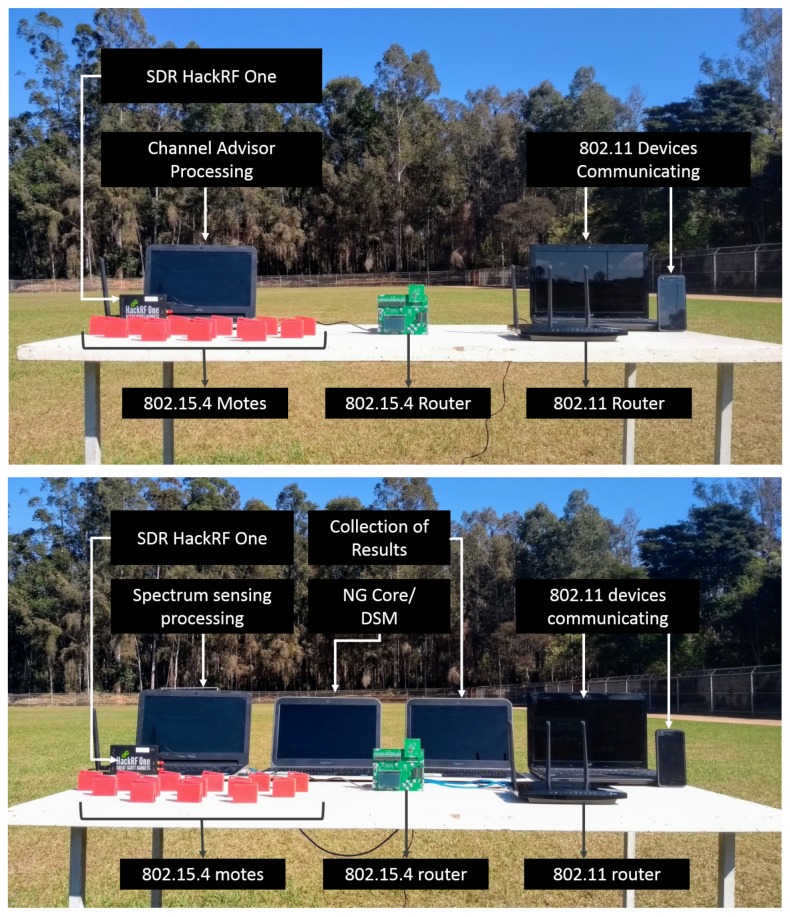
Experimental scenarios close to a soccer field to avoid interference to other devices outside experiment. (**a**) Scenario without NG; (**b**) Cognitive radio system scenario interoperating with NG.

All IEEE 802.15.4 devices run a Contiki [76] operational system (OS) and communicate one another via a low power wireless personal area network (6LoWPAN) with constrained application protocol (CoAP) [77]. 6LoWPAN devices are typically battery-powered and have low processing capability. CoAP is an application protocol for constrained devices, since it is lightweight and low power consuming. Furthermore, Contiki is an event-driven embedded OS for IoT nodes that allow dynamic loading and unloading of individual programs and services. The 802.15.4 motes generate traffic at the data plane, continuously publishing temperature measurements to an IEEE 802.15.4 border router. The described scenario is depicted in Figure 9. We will first present and discuss Figure 9a scenario (in Section 5.2), in which manual coordination is applied, instead of NovaGenesis architecture. NG coordination is presented in Section 5.3.

**Figure 10:**
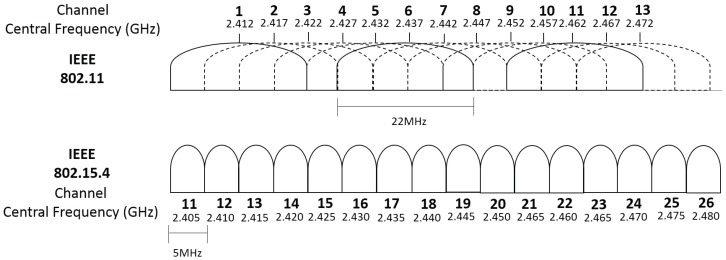
2.4 GHz ISM frequency bands of the 802.15.4 and 802.11 standards.

In a worst interference situation, the Wi-Fi AP operates in the channel 7, which has central frequency of 2.442 GHz. The 802.15.4 network operates, at the same time, at the default channel 18, which has central frequency of 2.440 GHz. Those channels were chosen to ensure the interference of the signals transmitted, since they have coincident channel frequencies. Figure 10 presents the frequency of each channel for 802.11 and 802.15.4. The 802.11 AP transmits at 22 dBm, while the 802.15.4 motes transmits at 5 dBm.

### 5.2. Results for Cognitive Radio System

The first test scenario consists of running the Channel Advisor to indicate the best network channels, without using the NovaGenesis to automatically coordinate all devices. We check the influence of 802.15.4 in the 802.11 operation and opposite situation.

#### 5.2.1. Evaluation of IEEE 802.15.4 Interference in the IEEE 802.11 Operation

**Figure 11:**
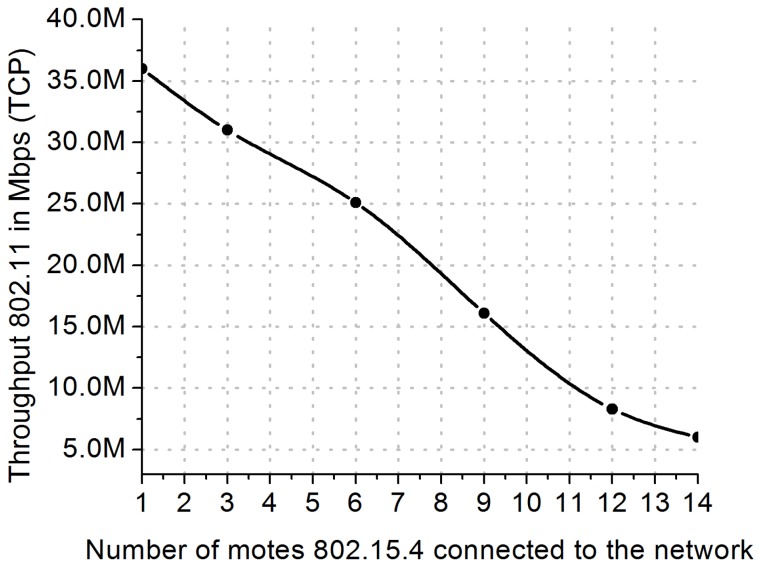
Variation in IEEE 802.11 throughput as new 802.15.4 motes are added to the network.

This scenario, as illustrated in Figure 9a, consists of evaluating the IEEE 802.11 throughput while IEEE 802.15.4 causes interference. To estimate the motes interference in the data plane of IEEE 802.11 standard, TCP throughput was evaluated when a new mote came into the network. Figure 11 reports the throughput decrease for each new mote connected to the IEEE 802.15.4 network, when IEEE 802.15.4 operates at the channel 18 and IEEE 802.11 at the channel 7. Figure 11 was measured in a scenario without the NovaGenesis control plane. The reported results indicate fast dropping of IEEE 802.11 throughput when IoT network size increases. This result justifies all the efforts being made to increase and dynamically manage ISM band allocations. Changing IoT nodes to other RF channels is an effective way to deal with the increasing of IoT motes in a network. However, there are limits to what can be done with channel switching, which indicates that the radio technologies themselves (physical and link layers) need to evolve to support the high congestions of spectrum usage in the 2.4 GHz ISM band.

We turned ON the Channel Advisor to sense IEEE 802.11 channels, verifying the best one to enter into operation. The throughput of the IEEE 802.11 was 6.1 Mbps with the interference of fourteen 802.15.4 motes. The Channel Advisor recommended operation at the channel 11. After switching the operation of the IEEE 802.11 router to the channel 11, the new IEEE 802.11 TCP throughput was 34.86 Mbps, i.e., an increase of 4.7 times in the throughput, by making the rate next to the non-interference condition.

#### 5.2.2. Evaluation of IEEE 802.11 Interference in the IEEE 802.15.4 Operation

It is possible to evaluate the interference of the Wi-Fi network operation when geographically close to an IEEE 802.15.4 network. Without the Wi-Fi AP interference, the IEEE 802.15.4 CoAP throughput was 1120 bps. After the Wi-Fi simultaneously operation at the channel 7, the 802.15.4 CoAP throughput decreased to 520 bps. The Channel Advisor automatically switched the 802.15.4 operation from the channel 18 to the 21. The new CoAP throughput was 1270 bps, a value close to the non-interference condition.

### 5.3. Results with NovaGenesis Control Plane

The second scenario consists of running the Channel Advisor to optimize wireless network device channels through NovaGenesis in order to automatically software-control their operation under the umbrella of SLAs. The thesis we want to proof is NovaGenesis will perform similarly to the Channel Advisor, but including the key design dimensions of Table 1. The experimental scenario with NovaGenesis is connected to a Sensing Cell through ZMQ socket as illustrated in Figure 7. In this way, the Sensing Cell can be geographically distant from the NG Core. In the next Subsections, we will follow Figure 8 steps, proofing NG concept for DSM.

#### 5.3.1. Exposition and Discovery of DSM Services

**Figure 12:**
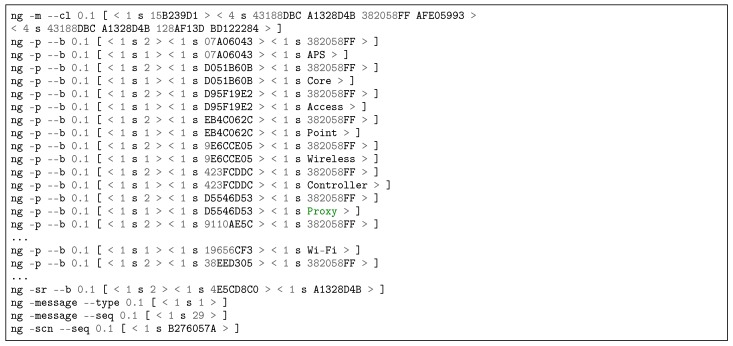
Log of APS exposition to enable RMS discovery of this access point proxy/control service.

**Figure 13:**
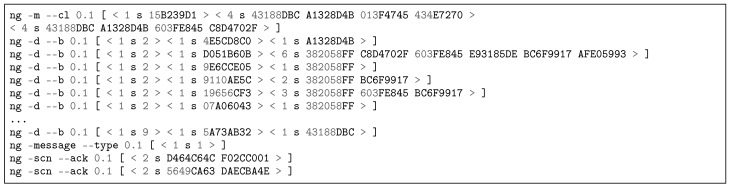
Log of the response from HTS to a RMS query about possible DSM peers.

Figure 12 contains a partial reproduction of the log of an APS exposition message published to RMS via NRNCS. The command line ng -m –cl is used for forwarding/routing [75]. The command line ng -p –b is used to publish name bindings, therefore exposing APS keywords to RMS, e.g., the key 19656CF3 is the hash of the word “Wi-Fi”. This log is a print of Transaction 1b in Figure 8. Besides APS, SSS, RMS and PGCS expose their names. In the discovery process, RMS subscribes keywords from the other DSM services. Figure 13 depicts a partial log of a NRNCS response to a RMS search (Trans. 2c in Figure 8). Name bindings inform about possible peers that will try to establish service contracts in the next step of DSM self-organizing approach. The command line ng -d –b is employed to deliver name bindings.

#### 5.3.2. Dynamic Contracting of DSM Services

**Figure 14:**
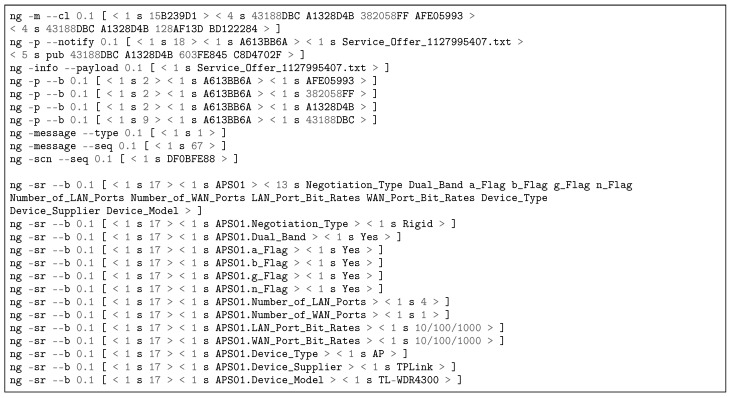
Log of APS service offer to RMS.

**Figure 15:**
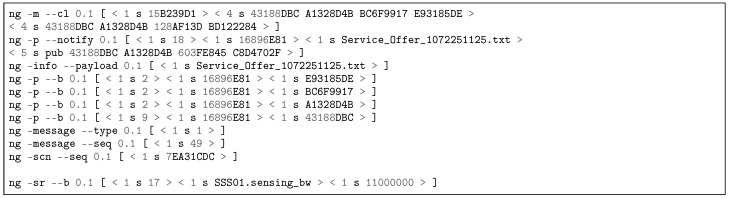
Log of SSS service offer to RMS.

Figure 14 reproduces APS service offer to RMS. The pub/notify command line (ng -p –notify) contains the name binding < 1 s A613BB6A >< 1 s Service_Offer_1127995407.txt >, which links the SVN A613BB6A to the .txt file containing the service offer. After the blank line, the features of the access point TL-WDR4300™ are detailed to RMS. The ng -info –payload informs the name of the .txt file to be stored at APS input/output (IO) folder in Linux operating system (OS). Figure 15 depicts SSS service offer to RMS, as illustrated in Figure 8 Transaction 3b. The SSS01.sensing_bw informs the Channel Advisor sensing bandwidth. All messages have an integrity check field (ng -scn –seq 0.1 [ < 1 s DF0BFE88 > ]) which allows to determine whether messages have been tampered with in transit or not. In all service offer cases, a service acceptance object is published by RMS and notified to APS/SSS/PGCS to inform RMS accordance. Both logs demonstrate NovaGenesis ability to represent other systems inside its environment, enabling proxying of any kind of legacy services/applications.

#### 5.3.3. The Best Wi-Fi Channel Indication and Changing

**Figure 16:**
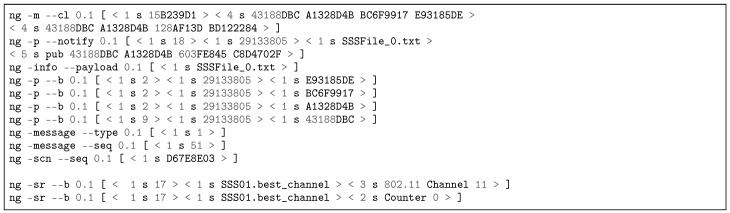
Log of SSS indicating for RMS the best channel to configure Wi-Fi access point in the region.

After the contract establishment, the DSM services can start operating as designed. Figure 16 reproduces the log of a SSS message sent to RMS indicating the best channel to be used to reduce interference with IEEE 802.15.4 sensor tags (Figure 8 Transaction 4b). RMS publishes the same indication to APS, which decides a channel change is required or not. Posterior, APS subscribes another file published with the same information and performs the change using SSH to connect to TL-WDR4300 OpenWRT, as illustrated in Figure 7. We measured the time required by the APS to subscribe a control file (SSSFile_0.txt) from NRNCS temporary cache as 8.666 ms.

#### 5.3.4. The Best IEEE 802.15.4 Channel Indication and Changing

SSS log changes when indication is related to IEEE 802.15.4 bandwidth. Instead of < 3 s 802.11 Channel 11 > ], SSS publishes < 3 s 802.15.4 Channel 23 > ], for instance. The remaining fields of Figure 16 are the same. In this case, RMS publishes to PGCS in order to change IoT nodes channels, including border router’. Differently from APS, PGCS employs Tunslip to send CoAP commands directly to the border router and sensor tags. We have also measured the time required by the PGCS to subscribe a control file (PGCSFile_1.txt) from NRNCS temporary cache as 9.106 ms.

#### 5.3.5. Evaluation of IEEE 802.15.4 Interference in the IEEE 802.11 Operation

This subsection reports the application of NG DSM for switching Wi-Fi AP channels. The throughput operating at channel 7 and interfered by a 802.15.4 network was 6.1 Mbps. As illustrated in Figure 8, SSS requests the Channel Advisor to sense IEEE 802.11 spectrum. After a best channel indication and RMS evaluation, APS has changed the Wi-Fi router to the channel 11. The same indication was automatically took by Channel Advisor without the use of NG architecture, for comparison purpose. After the channel change, the new TCP/IP/Wi-Fi throughput was 36.08 Mbps, a value close to the non-interference condition and similar to the Channel Advisor result.

**Figure 17:**
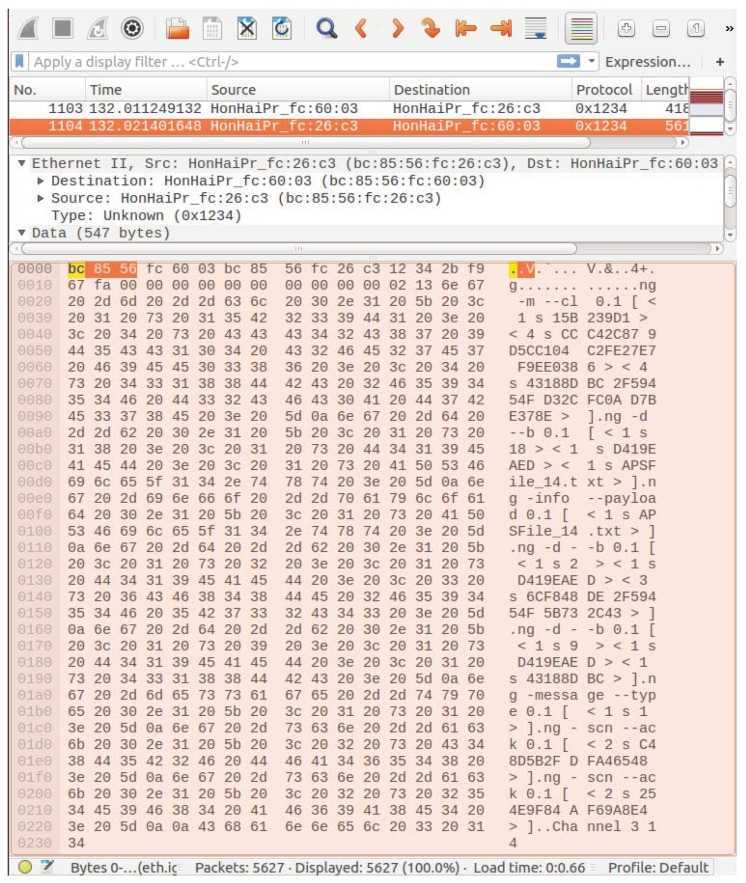
Capture of a NG message from RMS to APS using Wireshark™.

**Figure 18:**
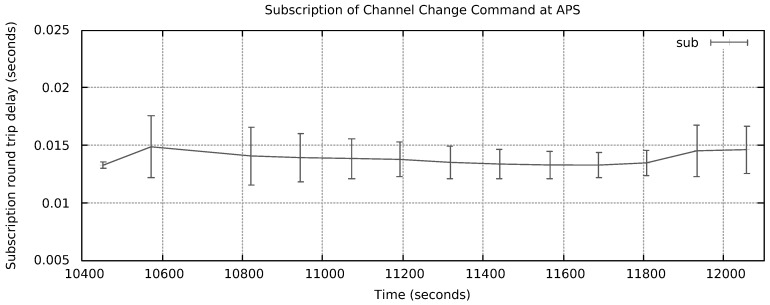
Mean round trip time of APS subscriptions for Wi-Fi channel changing command from PSS. Wi-Fi network delay between Host 1 and Host 2 is included twice.

**Figure 19:**
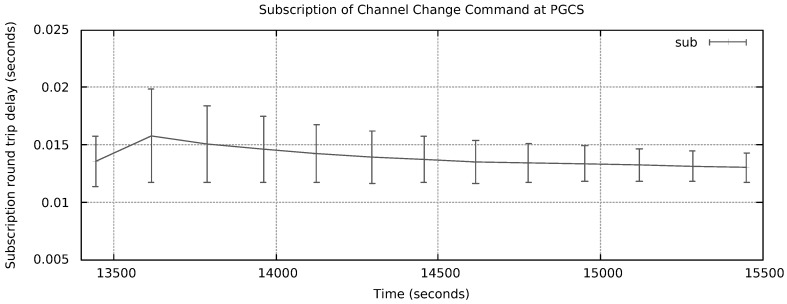
Mean round trip time of PGCS subscriptions for IEEE 802.15.4 channel changing command from PSS. Wi-Fi network delay between Host 1 and Host 2 is included twice.

In Figure 17, we reproduce a Wireshark™ capture of a NG message from RMS to APS requiring a channel change to channel 3. The control command is in the payload area of the message (latest bytes). For this test, the frame Type was configured to 0 × 1234, which is unknown for Wireshark. The mean round trip time (RTT) for subscribing such command messages at APS is depicted in Figure 18. Measurements started at time instant 10452.86 s and remained up to time instant 12056.72 s, i.e., 26.73 min. A total of 13 measurements were taken. The mean subscription RTT was 14.62 ms. The error bars present the worst and best results inside a 95% confidence interval, respectively. In contrast, Figure 19 reports mean RTT of IEEE 802.15.4 channel change messages subscriptions from PSS to Host 2 PGCS. Measurements started at time instant 13444.28 s and finished at 15450.32 s, i.e., 33.43 min. The mean subscription RTT was 13.05 ms. These delays demonstrate NG can be considered as an alternative architecture to deal with DSM of Wi-Fi/IoT in the unlicensed bands. A few milliseconds RTT look promising for control plane message subscription in a LAN environment.

#### 5.3.6. Evaluation of IEEE 802.11 Interference in the IEEE 802.15.4 Operation

The last test was focused on evaluating the benefits of our NovaGenesis-based approach to the IEEE 802.15.4 operation when interfered by a Wi-Fi network. The CoAP 802.15.4 throughput was 520 bps under 802.11 interference. PGCS has changed the 802.15.4 network to the channel 23, a channel free of Wi-Fi interference. The new 802.15.4 throughput increased to 1730 bps, a value even better than the obtained in the non-interference condition. Table 4 summarizes the results obtained for all performed tests. Significant throughput enhancements were obtained not only for Wi-Fi, but also for IoT nodes. Results for NovaGenesis are similar to the ones obtained with the Channel Advisor. This proves our NovaGenesis-based control plane represents an alternative to the status quo DSM technologies, which have limited support for the eight design dimension proposed in Table 1.

The obtained results proof-the-concept of our ICN-based, service-defined (APS and PGCS), named-data (PSS, GIRS and HTS) trustable IoT/Wi-Fi (service offers and acceptances) spectrum management approach (APS, RMS and SSS) for future wireless networks. NovaGenesis automates DSM for heterogeneous wireless networks, with delay of control packets exchanging in milliseconds range.

## 6. Discussion on Benefits and Open Challenges

This section returns to the design dimensions presented in Table 1, giving a summary of NovaGenesis benefits to the problem of trustable unlicensed spectrum management for IoT/Wi-Fi. Moreover, it points out open challenges for future developments. Table 5 summarizes our contributions for the control plane of new generation WSANs and IoT:

- D1—Our approach could sense any kind of wireless communication protocol in the HackRF One SDR operating bandwidth. It provides integrated IoT (IEEE 802.15.4) and Wi-Fi cognitive radio-based DSM. Our NG-based solution enable to increase network throughput and reduce interference of Wi-Fi access points in IoT nodes.
- D2—Secure exchange of spectrum sensing data via trustable DSM services. The novel security mechanisms proposed by NG, namely self-verifying naming, secure name resolution, trust network formation, contract-based operation and services reputation allows enhanced security at smart places. These mechanisms improve traditional security for IoT/FI/CR/5G, since it takes advantage of social behavior of devices and services.
- D3—NovaGenesis made possible name-based access and routing of spectrum sensing data, including network caching for efficient, distributed and coherent software-control (control plane) of smart environments.
- D4—Integration of software-defined control and operation [37,49]. The current SDN model (based on the OpenFlow protocol) is limited to configure forwarding tables at link layer switches. NG allowed broader configuration and management of physical devices via their software representatives (e.g., DSM services). Therefore, NG extends software-defined paradigm towards exposing hardware capabilities to spectrum management services, enabling “richer” orchestration of resources.
- D5—NovaGenesis includes support for the dynamic composition of control plane services based on semantic and context-awareness. It provides mechanisms for the complete service life-cycling. Quality of service (QoS) can be measured from the established contracts, enabling estimation of services reputation. Services with low quality can loose its contracts, losing reputation. QoS and reputation will be subject of future works. Dynamic composition is available for network data, control and management planes.
- D6—NovaGenesis provides increased expressiveness [16,45], when compared to current information architectures. NovaGenesis is not the unique architecture concerned to improve protocols expressiveness, XIA [16] is also an example that deals with this issue. However, to the best of our knowledge, we have first applied XIA for IoT in 2017 [78].
- D7—IoT/Wi-Fi devices, spectrum data and spectrum management services are accessed by self-verifying identifiers, enabling ID-consistent mobility and guaranteeing provenance of spectrum data [21,22].
- D8—NovaGenesis provides contract-based operation of spectrum management services. The control of IoT/Wi-Fi unlicensed band channels is service-defined, contract-based and trustable.

This paper extended Alberti et al. [33] scenario to deal with the dual-mode (Wi-Fi/IEEE 802.15.4) best channel indications from a Channel Advisor middleware. We plan to extend this DSM approach to other next generation WSANs, such as LoRa, Sigfox, NB-IoT, etc. Operation in licensed band is also an open issue. Furthermore, this article extended NovaGenesis control plane for CR/IoT/Wi-Fi. However, NG data plane is limited to Ethernet and Wi-Fi. We have been developing extensions for LoRa, ZigBee, passive-optical networks and IEEE 802.15.4. In other words, we have been developing a NovaGenesis over X (NGoX) adaptation layer, which will enable PGCS to interoperate with embedded NG versions, such as the embedded proxy/gateway service (EPGS) proposed in [4]. In this context, embedding NovaGenesis at GNU Radio, IoT nodes and access points are still in its infancy. We plan to introduce NovaGenesis in all devices of Figure 7, eliminating SSH, ZMQ, Tunslip and CoAP need. We indeed envision the need to improve spectrum sensing to a cooperative approach with NovaGenesis.

There is an inherent complexity behind the integration of so many ingredients, such as IoT, ICN, SDN/NFV, SOA, cognitive radio, dynamic spectrum management, etc. In this context, an open challenge is how to model the complexity behind existent/novel architectures in order to enable fair comparison. NovaGenesis brings to the core many ingredients usually found at the world wide web of the current Internet. For instance, distribute hash table (DHT) is typically implemented over TCP/IP. NovaGenesis implements DHT directly over link layer protocols, in the core. The same applies for MQTT, an IoT publish/subscribe approach implemented over TCP/IP. NovaGenesis implements pub/sub in the core, allowing any application to take advantage of this model. Therefore, to be fair, the same features should be present in both stacks being compared. It means we need to consider the overhead of application protocols of the Internet in the evaluation.

Another point is performance evaluation of ICN caches. Fair comparisons require complete scenarios, in which data/control plane information is transferred not to an unique client, but to many in a coherent and efficient way. It is in this type of situation that the new proposals are promising, taking advantage of ICN caches for coherent distributed deliveries. In summary, the evaluation of system performance should consider functionalities that are being covered, including aspects related to distributed coordination, data delivery and coherence of control plane. Methodologies for these aims provide interesting research opportunities and are topics for future research.

We also have plan to improve NovaGenesis performance by: (i) adding source coding to NG messages; (ii) exploring load balancing and multi-path routing; (iii) elasticity of NovaGenesis services; (iv) refinement of the prototype; (v) employ different hash code sizes to reduce overhead in NG messages; (vi) implement hierarchical multi-domain/level name resolution, network caching and name-based routing. These improvements promise a better performance when compared to the current solutions. For this reason, they will be the target of future work.

Scalability of route-by-name approaches with hierarchical networking caching is another important aspect to be evaluated in ICN-based approaches [65]. Moreover, our solution will require an evaluation regarding mobile nodes, such as the one performed by Siris et al. [68]. Reproducibility of experiments is another open issue, as well as to experiment with larger numbers of IoT nodes, Wi-Fi APs and spectrum sensing cells. Recent work on experimental facilities can help on improving such issues. Another future work is to prepare NovaGenesis scenarios to be tested in larger testbeds in Europe, USA and Brazil. A challenge is how to port NovaGenesis services to different hardware available in these experimental facilities.

For networks larger than a small campus network, more instances of spectrum sensors, best channel indicators and channel changing services will be required. The hardware part of our DSM solution can be scaled by adding more devices to the deployment. The software part of the our proposal can be scaled by employing elasticity of edge computing as proposed by Righi et al. [80]. Moreover, previous results obtained for NovaGenesis service-oriented, self-organizing, dynamic composable distributed architecture [4,11,33] indicate that it can deal with new software instances as the number of physical devices increases. However, a large scale experiment with hundreds/thousands of nodes is a quite challenging research task, which is being addressed by the authors as future works.

## 7. Conclusions

This paper presented, for the first time, a successful application of a future Internet architecture for the integrated (IoT/Wi-Fi) dynamic spectrum management of wireless devices (access points and sensor nodes) in a smart campus scenario, which can be extended to a larger scale. We have extended previous NovaGenesis services [33] to provide ISM unlicensed band best channel indications not only for IEEE 802.11, but also for IEEE 802.15.4 standard.

A spectrum sensing service was extended to expose dual-mode (Wi-Fi/IoT) best channel indication feature to other NovaGenesis dynamic spectrum management services. SSS interoperates with Channel Advisor, which has a GNU Radio implementation for determining energy level at Wi-Fi/IoT unlicensed bandwidths. A resource management service was extended to deal with this dual mode operation. A novel APS was developed to represent Wi-Fi APs towards establishing control plane contracts to a RMS instance. Existing PGCS was extended to represent IEEE 802.15.4 Texas Instruments cc2650 sensor tags and border routers. PGCS establishes control plane contracts to a RMS instance, making necessary protocol translations and encapsulations to change IoT nodes radio frequency channels.

In summary, our approach enables coordination of any group of devices operating with different wireless communication standards. The overall architecture was idealized, implemented and tested in a real scenario to proof two main hypotheses: (i) the Channel Advisor improves throughput when acting automatically in a specific wireless network standard; (ii) NovaGenesis obtains the same results, but automating DSM in a contract-based way for any group of wireless network standards. Both hypotheses were tested in open field and validated. Channel Advisor operating separated in one only network showed results that improved the 802.11 throughput in 4.71 times and the 802.15.4 throughput in 1.44 times. When checking the efficient of NovaGenesis acting automatically in both networks, the throughput improved in 802.11 was 4.91 times compared to the interfered rate and the 802.15.4 improvement was 2.33 times.

These results demonstrate NovaGenesis-based control plane can be seen as an alternative to the status quo DSM technologies, which have limited support for the previously reported design dimensions. The control packets subscription delays remained limited to a few milliseconds in the evaluated scenario, which are promising values for real applications. Future works include continuing extending this solution towards: (i) other next generation sensor and wireless networks, such as LoRa, Sigfox, NB-IoT, etc; (ii) integration with licensed bands for IoT; (iii) develop a NovaGenesis over X (NGoX) adaptation layer; (iv) embedding NovaGenesis at GNU Radio, IoT nodes and access points; (v) improve spectrum sensing to a cooperative approach with NovaGenesis; (vi) increase scalability and reproducibility of experiments.

## Figures and Tables

**Table 1:** Architecture design dimensions considered in this article.

Dimension	Description
D1	Dynamic cognitive radio-based spectrum management in licensed/unlicensed spectrum bands.
D2	Secure exchange of IoT control data via trustable services.
D3	Name-based access and routing of control data (spectrum sensing), including network caching.
D4	Software-defined control and operation.
D5	Dynamic composition of control services based on semantic and context-awareness, including complete service life-cycling.
D6	Improved support for architecture data and entities naming and name resolution.
D7	Identifier and locator splitting, meaning different names are used for identifying and locating data and services.
D8	Contract-based operation of control plane services.

**Table 2:** Related work on the next generation WSANs and IoT networks. Comparison with respect to: D1—Dynamic spectrum management with cognitive radio; D2—Secure exchange of control data via trustable services; D3—Named-control-data access and routing; D4—Software-defined control and operation; D5—Dynamic composition of control services; D6—Improved naming and name resolution for IoT; D7—Identifier/locator splitting for architecture entities; D8—Contract-based control plane.

	Dimensions							
Previous Work	D1	D2	D3	D4	D5	D6	D7	D8
Energy Harvesting Cognitive Radio Networking for IoT-enabled Smart Grid [40]	x							
A Secure IoT Management Architecture based on Information-Centric Networking [20]		x	x	x		x	x	
LASeR: Lightweight Authentication and Secured Routing for NDN IoT in Smart Cities [22]			x			x		
Spectrum Management for Proactive Video Caching in Information-Centric Cognitive Radio Networks [26]	x	x	x			x		
Spectrum-Availability based Routing for Cognitive Sensor Networks [41]	x							
A Case for ICN Usage in IoT Environments [27]			x			x		
A Comparative Study of MobilityFirst and NDN based ICN-IoT Architectures [28]			x			x		
A De-verticalizing Middleware for IoT Systems Based on Information Centric Networking Design [42]		x				x		
A Distributed ICN-based IoT Network Architecture: An Ambient Assisted Living Application Case Study [43]		x				x	x	
A Robust and Lightweight Name Resolution Approach for IoT Data in ICN [44]			x			x		
A Secure ICN-IoT Architecture [23]			x		x	x		
A Software-Defined Networking Framework to Provide Dynamic QoS Management in IEEE 802.11 Networks [37]	x			x				
A Cloud-Based Internet of Things Platform for Ambient Assisted Living [45]					x			
Coexistence of Wi-Fi and Heterogeneous Small Cell Networks Sharing Unlicensed Spectrum [46]	x							
Cognitive Radio-Enabled Internet of Vehicles: a Cooperative Spectrum Sensing and Allocation for Vehicular Communication [47]	x							
Consumer Oriented IoT Data Discovery and Retrieval in Information Centric Networks [48]			x			x		
CORAL-SDN: A Software-Defined Networking Solution for the Internet of Things [49]				x				
Cross-Technology Wireless Experimentation: Improving 802.11 and 802.15.4e Coexistence [50]	x			x				
Development of Measurement Techniques and Tools for Coexistence Testing of Wireless Medical Devices [51]	x			x				
Distributed Channel Allocation and Time Slot Optimization for Green Internet of Things [52]	x							
Dynamic Spectrum Access for Internet of Things Service in Cognitive Radio-Enabled LPWANs [53]	x							
Efficient Methods of Radio Channel Access using Dynamic Spectrum Access that Influences SOA Services Realization – Experimental Results [54]	x							
Energy-Efficient Channel Handoff for Sensor Network-Assisted Cognitive Radio Network [39]	x							
Experimental Study of Coexistence Issues Between IEEE 802.11b and IEEE 802.15.4 Wireless Networks [55]	x							
Adaptive Radio Channel Allocation for Supporting Coexistence of 802.15.4 and 802.11b [56]	x			x				
The SDN Approach for the Aggregation/Disaggregation of Sensor Data [9]				x				
Performance and Challenges of Service-Oriented Architecture for Wireless Sensor Networks [57]				x				
ISI: Integrate Sensor Networks to Internet with ICN [58]			x		x			
Software-Defined Network Virtualization: An Architectural Framework for Integrating SDN and NFV for Service Provisioning in Future Networks [59]		x		x	x			
Networking Named Content [18]			x			x	x	
A Survey of Information-Centric Networking [19]			x			x	x	
A Survey of Information-Centric Networking Research [29]			x			x	x	
Named Data Networking [30]			x			x	x	
Efficient Proactive Caching for Supporting Seamless Mobility [60]			x		x			
Efficient Information Lookup for the Internet of Things [61]						x	x	
Cloud Computing for Global Name-Resolution in Information-Centric Networks [62]			x		x			
Network of Information (NetInf) - An Information-centric Networking Architecture [17]			x			x	x	
XIA: Efficient Support for Evolvable Internetworking [16]			x		x	x	x	
Prototyping the Recursive Internet Architecture: the IRATI Project Approach [31]		x	x		x	x	x	x
Developing information networking further: From PSIRP to PURSUIT [32]			x			x	x	

**Table 3:** NovaGenesis concepts [3].

Concept	Description
Name	Symbols that denote an existence in natural language.
Identifier	An unique name that unambiguously identify an existence in a certain scope.
Locator	A name that denotes a certain position or point of attachment in a certain space, giving notation of distance to other points in the same space.
Name Binding (NB)	An entity that link names.
Process	An instance of a computer program running in an operating system that has Blocks and Actions internally.
Block	An internal component of a Process that contains many Actions.
Action	An internal component of a Block that implements its functioning.
Message	The protocol data unit (PDU) for NovaGenesis information exchange.
CommandLine	Each command line describes an Action to be executed at the destination and its parameters.
Service	The same than a Process.
Hash Table (HT)	An instance (Block) that implements a hash table data structure.
Gateway (GW)	A Block responsible to exchange messages inside a process.
Proxy/Gateway (PG)	A Block responsible to exchange messages externally a process.
Hash Table Service (HTS)	A distributed hash table build with HT Blocks.
Generic Indirection Resolution Service (GIRS)	Responsible to select the proper HTS to store name bindings and content.
Publish/Subscribe Service (PSS)	Responsible for the rendezvous of publishers and subscribers.
Proxy/Gateway/Controller Service (PGCS)	Encapsulation of messages for link layer transport, representative of things and software-controller of their configurations.

**Table 4:** Throughput results before and after changing the channel.

Description	Before	After	Throughput Gain
802.15.4 interference in 802.11 without NG	6.1 Mbps	34.86 Mbps	471%
802.11 interference in 802.15.4 without NG	520 bps	1270 bps	144%
802.11 interference in 802.15.4 with NG	6.1 Mbps	36.08 Mbps	491%
802.15.4 interference in 802.11 with NG	520 bps	1730 bps	233%

**Table 5:** NovaGenesis DSM for IoT/Wi-Fi. D1— Dynamic spectrum management with cognitive radio; D2—Secure exchange of control data via trustable services; D3—Named-control-data access and routing; D4—Software-defined control and operation; D5—Dynamic composition of control services; D6—Improved naming and name resolution for IoT; D7—Identifier/locator splitting for architecture entities; D8—Contract-based control plane.

	Approach Taken	Benefits for Smart Environments	Contributions to State-Of-The-Art
D1	Protocol-agnostic best channel indication based on the radio frequency energy of operational channels. Exposition of spectrum sensing and channel control services in IoT and Wi-Fi.	Programmability [9], improved expressiveness [16,17,18,45], flexibility and cohesive integration to IoT.	ISM band spectrum sensing and best channel indication as a service. Dual mode (Wi-Fi/IEEE 802.15.4) operation.
D2	Asynchronous and distributed access to control data using self-verifying names [21] and name-based forwarding, routing and delivery of spectrum control data.	Coherence of control actions, security (integrity) of control messages [23], provenance of control data [22]. All these features are determined in terms of control file SVNes.	First application of ICN paradigms to control and management of DSM in IoT/Wi-Fi.
D3	Access to control files is given by name bindings published in NRNCS. Representatives of controlled devices (PGCS and APS) are notified and subscribe about control files. Queries follow a path to the NRNCS instance. Control files are delivered by HTS directly to PGCS and APS.	In-network name-based coordination of services [20], in-network caching of control files [20], asynchronous/coherent IoT/Wi-Fi command execution, name-based security [21], efficiency of control dissemination [58], unbounded namespaces [58].	A convergent ICN, CR and SOA approach for WSANs and IoT control plane. Suarez et al. [20] apply ICN for IoT management, including registration and discovery of devices, command execution and retrieval of measured data. Besides these features, our work integrates ICN with SOA and CR, advancing life cycle of control services.
D4	An alternative to OpenFlow SDN is employed to chance configurations at Wi-Fi access points and IEEE 802.15.4 sensor tags. This alternative is generic, flexible and adequate to support command execution on IoT/Wi-Fi devices.	Flexibility, self-configuring, improved controllability and management, support for dynamic QoS [37,49].	An alternative to SDN/NFV for IoT. CORAL-SDN embeds a programmable data plane at IoT nodes [37,49]. It also leverages a modified controller to support IoT nodes topology control, routing and flow establishment, as well as data collection. However, CORAL-SDN neither covers DSM, nor employs ICN at the control plane.
D5	To apply SOA principles for IoT/WSAN control plane. DSM and IoT services can expose their features, search for partners and form trust networks based on a service level agreement.	Context-awareness, contract-based operation, integration of heterogeneous devices and middlewares, self-organization and coordinated orchestration [45].	In [45], discovery and control services are developed, but none related to coexistence of RF signals. An alternative to the IP-based WSN SOA architecture proposed in [79]
D6	Support for spectrum data, control and services naming and name resolution via the hierarchical, distributed, NRNCS.	The improved expressiveness allows DSM/IoT services to express their keywords, names (natural language and self-verified) and service offers to possible peers.	Besides ICN and SCN provided by XIA [16], NG employs SOA and contract-based operation. In addition, XIA has not been applied for cognitive radio yet.
D7	To decouple entities identifiers (IDs) from locators (Locs), enabling direct entities access via IDs, independently of their locations (LOCs).	Mobility without identity loss [36]. Perennial identification of data, devices and services.	In [20], ID/Loc splitting is provided to IoT management. Our work provides a generic ID/Loc splitting approach to all architectural entities, including devices and services.
D8	A novel service-defined approach to allow exposing best channel indication (or spectrum sensing) features to DSM services.	Trust-ability, security, reputation of control services.	An ecosystem of trustable services for IoT/WSAN control plane. Suarez et al. [20] also provides ICN-based SLAs. A difference is that NG binds contract-names to service names, improving security.
